# Experimental Research on Viscosity Characteristics of Grouting Slurry in a High Ground Temperature Environment

**DOI:** 10.3390/ma13143221

**Published:** 2020-07-20

**Authors:** Jiandong Niu, Bin Wang, Chu Feng, Kang Chen

**Affiliations:** School of Civil Engineering, Central South University, Changsha 410000, China; binwang@csu.edu.cn (B.W.); fengchu@csu.edu.cn (C.F.); 184812184@csu.edu.cn (K.C.)

**Keywords:** high ground temperature environment, grouting slurry, viscosity characteristics, experimental research, function fitting method

## Abstract

The grouting method is a technical means to prevent and control the thermal damage of the tunnel with high ground temperature in the underground hot water area, and the viscosity characteristic of the slurry is the key factor of grouting treatment. When grouting in high ground temperature geological conditions, the slurry inevitably has both time-varying and temperature-varying characteristics of viscosity in the process of filling high-temperature fissures and plugging geothermal water. At present, the research on the viscosity characteristics of slurry at high temperature is rarely reported in the literature. In this paper, laboratory tests were carried out to measure the time-varying viscosity of ordinary cement slurry; cement-sodium silicate slurry, widely used in engineering; and high ground temperature grouting slurry (HGTGS), independently developed by our research group, at different preheating temperatures (20, 40, 60, 80 °C). The viscosity function curves of the three kinds of slurry were obtained by function fitting method, and the viscosity variation law of slurry was analyzed. The study found that the time-varying process of viscosity of the cement-sodium silicate slurry and the HGTGS in specific temperature conditions can be divided into two stages: The slow rising period of viscosity and the rapid rising period of viscosity. Whereas, the time-varying process of viscosity of ordinary cement slurry in specific temperature conditions includes only one stage of the slow rising period of viscosity. The viscosity of ordinary cement slurry and cement-sodium silicate slurry increases with the increase of temperature, while the viscosity of the HGTGS decreases with the increase of temperature. There are corresponding viscosity time-varying equations for the three kinds of slurry in different temperature conditions. The viscosity time-varying equation of the ordinary cement slurry accords with the linear function form, and the viscosity time-varying equation of the cement-sodium silicate slurry accords with the power-law function form. The viscosity time-varying equation of the HGTGS conforms to the exponential function form. On this basis, the unified description equation of the viscosity characteristics of grouting slurry in high ground temperature and the applicability of different types of grouting materials are obtained. This study has a certain reference value and guidance for theoretical analysis, numerical simulation and engineering application of grouting in high ground temperature environment.

## 1. Introduction

**Figure 1:**
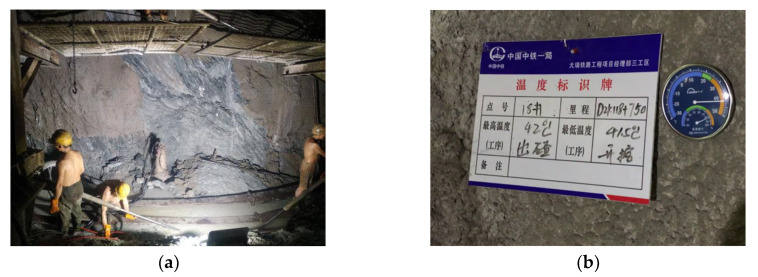
Tunnel with high ground temperature encountered in tunnel construction at present. (**a**) The palm face of a tunnel with high ground temperature; (**b**) the rock temperature of a tunnel with high ground temperature is 42 °C.

In recent years, tunnel construction has gradually extended to remote mountainous areas and complex geological environments, leading to the emergence of more high ground temperature tunnels (shown in Figure 1) [1,2]. Grouting is a method to prevent and control the thermal damage of a tunnel with high ground temperature in an underground hot water area, and the viscosity characteristic of slurry is the key factor of the grouting treatment. The engineering practice shows that the viscosity of the slurry is not only related to the transport time of the slurry, but also related to the external ambient temperature of the slurry. Because the surrounding rock temperature and underground hot water temperature of a high geothermal tunnel are often between 30 to 80 °C, during water plugging and reinforcement by grouting in the surrounding rock of a high geothermal tunnel, the slurry at normal temperature is first running in the borehole and heated by hot water and rock wall in the hole. Then, entering the crack in the surrounding rock will be heated rapidly, so the process of slurry transportation is the process of slurry being heated step-by-step. It can be seen that, in the high ground temperature tunnel grouting construction, the viscosity of the slurry will inevitably be affected by the environmental temperature in the fissures and grouting time. If the time-varying and temperature-varying characteristics of viscosity of the slurry are not taken into account, it is bound to seriously affect the effect of grouting. In addition, the viscosity characteristics of grouting materials must also be considered in the establishment of grouting theory and numerical calculation in a high ground temperature environment. Therefore, it is of great significance to study the viscosity characteristics of grouting materials at high temperature.

The grouting construction in high ground temperature geological conditions promotes the research and development of grouting materials at high temperature. The key to the research of grouting materials at high temperature is the influence of curing temperature. In particular, curing temperature has an important influence on the hydration process and hydration results of cement-based grouting materials. There are obvious differences in the hydration degree of cement, the composition and structure of hydration products and the macroscopic physical and mechanical properties of the stone body formed after cement slurry setting and hardening in different curing temperature conditions [3]. 

In terms of the reaction mechanism of grouting materials at high temperature, Termkhajornkit et al. [4] have studied the effect of curing temperature on the hydration rate of cement. The results show that, in the condition of high temperature, the dissolution of cement is accelerated, the hydration reaction is intense and the precipitation and lap rate of hydration products increase. Zakoutsky et al. [5] and Kumar et al. [6] have studied the hydration exothermic process of cement in different curing temperatures. The results show that the higher the temperature, the earlier the hydration exothermic peak appears, and the higher the peak value. Gallucci et al. [7] believe that the increase of curing temperature will accelerate the early hydration rate of cement, but does not affect the final hydration degree. The formation of the internal microstructure of cement-based grouting materials is mainly in the early stage of hydration, during which the genes that determine the pore structure, physical and mechanical properties and durability of the paste will be formed. Mineral admixture has a significant effect on the hydration process of cement-based grouting materials in the condition of high temperature. Narmluk et al. [8] found that, in the condition of high temperature curing, fly ash can promote the hydration of cement in the early stage and delay the hydration in the later stage. Zhang et al. [9] studied the early hydration and hardening process of slurry mixed with silica fume in different curing temperatures. The results show that the increase of curing temperature can greatly accelerate the early hydration process, and with the increase of silica fume content, the hydration process is also significantly accelerated. In addition, Zhang et al. [10] also studied the hydration process of multi-component composite cementitious materials mixed with fly ash, silica fume and slag at high temperature, and found that increasing curing temperature can accelerate the early hydration process. 

In the study of the properties of grouting materials at high temperature, Shi et al. [11] studied the basic properties of cement slurry, such as setting time, viscosity, water precipitation rate and compressive strength at different temperatures. the results show that, with the increase of temperature, the initial and final setting time shortens, the viscosity increases and the water precipitation rate decreases. At lower temperature, the compressive strength of slurry stone body increases with the increase of temperature and age, whereas at higher temperature, the later strength of slurry stone body decreases with the increase of temperature. Wang et al. [12], Sajedi et al. [13] and Al-Gahtani [14] have also found a similar rule in the study of the effect of curing temperature on the strength development of slurry stone body. Dai et al. [15] have studied the effect of temperature on paste structure at low constant shear rate. The results show that with the increase of temperature, the elasticity, plasticity and viscosity of paste increase, initial shear stress and dynamic yield stress increase, energy storage modulus and static yield stress increase. Liu et al. [16] measured the initial and final setting time of VCH (vinyl cyclohexene) cement-based grouting material at different temperatures by using the mortar setting time tester, and found that the initial and final setting time of the slurry shortened greatly with the increase of temperature. It is pointed out that, in practical application, the mixture ratio of the material should be reasonably selected according to the field temperature and groundwater temperature. Mineral admixture is an important component of composite grouting materials. In terms of the effect of slag on the properties of cement-based grouting materials at high temperature, Chen et al. [17] found that slag powder plays an important role in improving the fluidity and stability of cement slurry, avoiding premature setting and hardening of cement slurry at high temperature, and improving the compressive strength of cement paste in the later stage. Abd-El Aziz et al. [18] tested the pastes with slag substitution rates of 10%, 20%, 30%, 40% and 50%, respectively, and the results showed that the strength of all cement pastes increased with the increase of curing temperature. Sajedi [19] also found that the compressive strength of cement paste mixed with slag in a high temperature curing condition is higher than that in a standard curing condition. 

According to the viscosity characteristics of slurry, Zhu et al. [20] found that the viscosity of cement slurry changed little in the process of grouting, while the viscosity of cement-sodium silicate slurry increased rapidly with the increase of time. Yang et al. [21] and Du et al. [22] have done a lot of research on the variation law of the viscosity of all kinds of slurry, and proved that the viscosity of the slurry has time-varying characteristics, but the specific law of the time-varying viscosity of the slurry has not been deeply studied. Ruan [23,24] and Wang et al. [25] also proved that the viscosity of the slurry has time-varying characteristics, and its law accords with the exponential function. Li et al. [26,27] carried out laboratory tests on the quick-setting slurry commonly used in dynamic water grouting in underground engineering, fitted and analyzed the test data, and obtained a time-varying equation of the viscosity in different water:cement ratios of cement slurry and different slurry mixing volume ratios. Liu et al. [28] considered the time-varying characteristics of slurry viscosity, deduced the slurry diffusion equation based on capillary permeability theory, and compared and analyzed the difference of the pressure of the slurry on the segment with and without considering the time-varying characteristics of slurry viscosity. Zhang et al. [29] and Zhang et al. [30], based on the non-uniformity of the spatial distribution of viscosity of quick-setting slurry, established a grouting diffusion model considering the temporal and spatial variation of slurry viscosity. Hou et al. [31] proposed a porous media slurry diffusion model considering the variation of viscosity with time based on the Bingham fluid constitutive model, and verified the grouting diffusion model by numerical simulation. Zuo et al. [32] studied the effect of water:cement ratio on the viscosity of double-liquid cement-based grouting materials. The results show that the viscosity of single slurry decreases with the increase of water:cement ratio, and when the two kinds of slurry are mixed, the viscosity appears inflection point. Xie et al. [33] found that the apparent viscosity of fresh paste decreased with the increase of the amount of bentonite slurry. Zhang et al. [34] used the MATLAB program developed to study the flow of cement slurry in the concentric ring and determine the slurry viscosity distribution in the grouting area. Assefa et al. [35] established an empirical model of the relationship between slurry viscosity, solid volume fraction, maximum solid volume fraction, average particle size and uniformity coefficient by studying the viscosity of multi-particle Bingham slurry at high solid concentration. Liu et al. [36] studied the influence of the time-varying characteristics of slurry viscosity on grouting pressure and diffusion radius, deduced the grouting diffusion equation of deep-buried rock cracks and verified it by experiments. Zhang et al. [37] proposed a step-by-step calculation method to describe the grouting process by considering the non-uniform distribution of slurry viscosity in the grouting area, studied the distribution of slurry viscosity in the grouting area, and verified the effectiveness of the step-by-step calculation method through fracture grouting test. Campbell et al. [38] proved that the ratio of infinite shear viscosity to zero shear viscosity conforms to power law by using the Bingham rheological fluid model based on energy dissipation. Blissett et al. [39] established a model which can predict the relative viscosity of fly ash slurry as a function of concentration and shear rate with high precision. Xiao et al. [40] studied the rheological properties of coal water slurry with different concentrations at different temperatures. It was found that the Bingham plastic viscosity of coal water slurry increased with the increase of concentration and decreased with the increase of temperature. 

The above reports mainly focus on the effect of curing temperature on the hydration of cement-based grouting materials and the macroscopic physical and mechanical properties of slurry body. The research on the viscosity characteristics of slurry is only limited to the time-varying characteristics of slurry viscosity and the non-uniformity of spatial distribution of slurry viscosity in the process of grouting at room temperature, but there are few reports on the viscosity characteristics of slurry at high temperature. Therefore, it is necessary to make an in-depth exploration and study on the viscosity characteristics of grouting slurry at high temperature.

In this paper, according to the field conditions of a typical water-rich and high ground temperature tunnel, the high temperature environment is simulated in the laboratory. Using ordinary cement slurry, cement-sodium silicate slurry widely used in engineering and the high ground temperature grouting slurry (HGTGS) independently developed by our research group, the viscosity tests of slurry at different temperatures were carried out. The function fitting method is used to fit and analyze the test data. The mathematical analytical expression of the fitting curve is carried out. The viscosity time-varying equations of three kinds of slurry at different temperatures are established. The time-varying and temperature-varying characteristics of the viscosity of three kinds of slurry are revealed. This study can provide necessary guidance for the establishment of grouting theory, numerical calculation of grouting and engineering application of grouting in high geothermal geological conditions.

## 2. Materials and Methods 

### 2.1. Experimental Materials

#### 2.1.1. Cement

The cement used in the experiment was P.O.42.5 ordinary Portland cement. The major chemical constituents and physical properties of the cement are shown in Table 1. The quality of cement conformed to the standard of general Portland cement [41].

#### 2.1.2. Sodium Silicate Slurry

Sodium silicate slurry is sold on the market, its Baume degree is 40 °B é, and its modulus is 3.0. According to the proportion of sodium silicate slurry, which is often used in engineering, the original sodium silicate slurry was diluted to prepare a slurry with Baume degree of 25 °B é.

#### 2.1.3. The HGTGS

**Figure 2:**
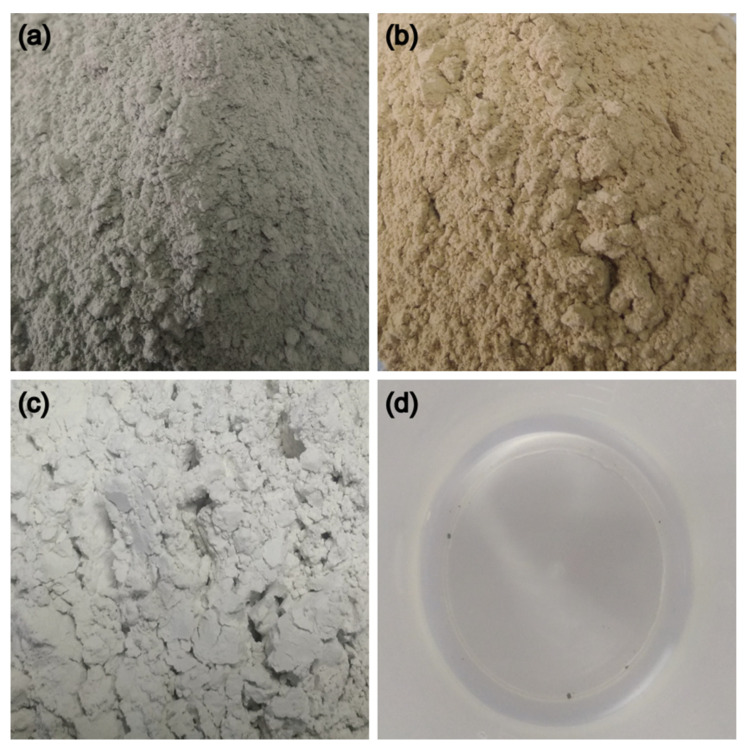

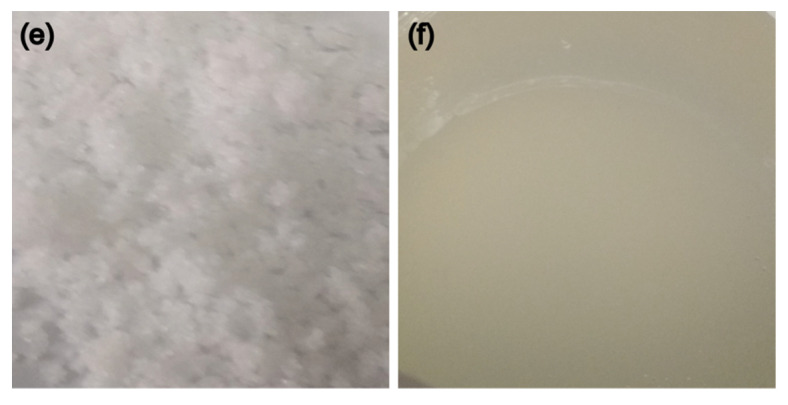
The main components of the high ground temperature grouting slurry (HGTGS). (**a**) The main components of group agent A. (**a**) Ordinary Portland cement; (**b**) bentonite; (**c**) slag powder; (**d**) tap water. (**b**) The main components of group agent B. (**e**) Modified additives; (**f**) sodium silicate slurry.

The HGTGS was made from the mixture of group agent A and group agent B. The main components of group agent A and group agent B are shown in Figure 2. The chemical compositions of the HGTGS are shown in Table 2. The main properties of the HGTGS are shown in Table 3 and Table 4. The viscosity characteristics of the HGTGS are representative.

It can be seen from Table 4 that the 3 days compressive strength and rupture strength of stone body of the HGTGS gradually increase with the increase of temperature, and when the temperature is 80 °C, its compressive strength and rupture strength increase by 88.37% and 76.47%, respectively, compared with those at 20 °C. The 7 days compressive strength and rupture strength of stone body of the HGTGS increase at first and then decrease with the increase of temperature, and reach the maximum at 60 °C. At this time, its compressive strength and rupture strength increase by 61.28% and 36.55%, respectively, compared with those at 20 °C. The 28 days compressive strength and rupture strength of stone body of the HGTGS increase at first and then decrease with the increase of temperature, and reach the maximum at 60 °C. At this time, its compressive strength and rupture strength increase by 30.86% and 35.98%, respectively, compared with those at 20 °C. The 56 days compressive strength and rupture strength of stone body of the HGTGS increase at first and then decrease with the increase of temperature, and reach the maximum at 60 °C. At this time, its compressive strength and rupture strength increase by 22.05% and 21.40%, respectively, compared with those at 20 °C. The 90 days compressive strength and rupture strength of stone body of the HGTGS increase at first and then decrease with the increase of temperature, and reach the maximum at 60 °C. At this time, its compressive strength and rupture strength increase by 20.08% and 21.22%, respectively, compared with those at 20 °C. The 7, 28, 56 and 90 days compressive strength and rupture strength of stone body of the HGTGS increase at first and then decrease with the increase of temperature. They both reach the maximum when the temperature is 60 °C, and then begin to decrease after 60 °C. The reason may be that at lower temperature, the gel product is formed slowly in the gel reaction process of slurry and the gel product has enough time to spread, precipitate and overlap in an orderly manner, resulting in a dense structure and low porosity, which improves the strength of stone body of slurry. On the contrary, at higher temperature, the gel product is formed faster in the gel reaction process of slurry, and a large number of gel products will precipitate and overlap disorderly in a short time, resulting in a large porosity of the structure, which reduces the strength of stone body of slurry.

The above results show that the increase of temperature can improve the early strength of stone body of slurry, but it is disadvantageous to the late strength development of stone body of slurry. The HGTGS will exert its best strength performance at the ambient temperature of 60 °C. In addition, in the environment with a temperature below 60 °C, the late strength of stone body of the HGTGS will gradually increase and stabilize at a higher strength value; while at an ambient temperature of 80 °C, the early strength of stone body of the HGTGS will be high and its late strength will be low, but the late strength will also be stabilized at a higher strength value. Therefore, in practical engineering application, the HGTGS can meet the needs of late strength and long-term stability of grouting materials in the water plugging engineering in high geothermal geology.

### 2.2. Experimental Instrument

**Figure 3:**
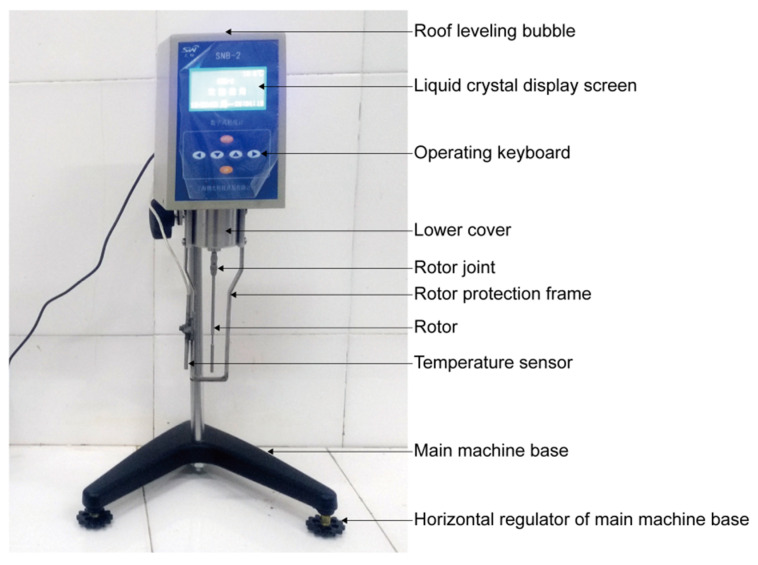
The SNB-2 microcomputer digital display viscometer.

The viscosity of slurry was tested by the Chinese SNB-2 microcomputer digital (Shanghai, China) display viscometer (Figure 3), which uses microcomputer control technology to digitally process the data detected by the sensor. The measuring range of the viscometer was 0.001–2000 Pa·s. When measuring, the viscometer can display the viscosity and temperature of the tested slurry on its display screen in real-time. In addition, through the data communication port of the viscometer, it can be connected with the personal computer, so that the test results can be saved and recorded in the computer.

The SNB-2 microcomputer digital display viscometer uses a motor to drive the rotor to rotate at a constant speed through variable speed. When the rotor rotates in the slurry, the slurry will produce the viscosity torque acting on the rotor. The greater the viscosity of the slurry, the greater the viscosity torque. On the contrary, the smaller the viscosity of the slurry, the smaller the viscosity torque. The viscosity torque acting on the rotor is detected and picked up by the sensor, and the viscosity of the tested slurry is obtained after computer processing.

### 2.3. Experimental Method

**Figure 4:**
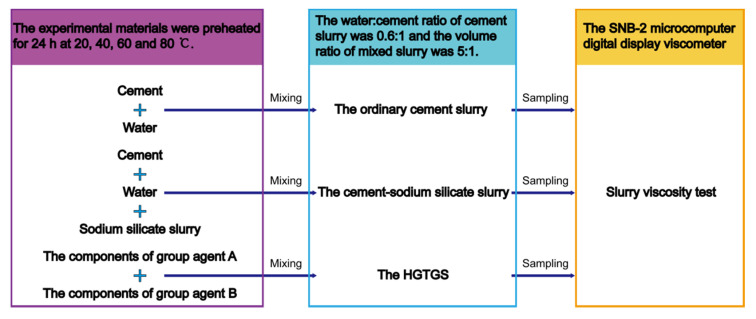
Experimental method.

The experimental method of this study is shown in Figure 4, which mainly included three parts: Materials preheating, slurry preparation and viscosity test.

**Figure 5:**
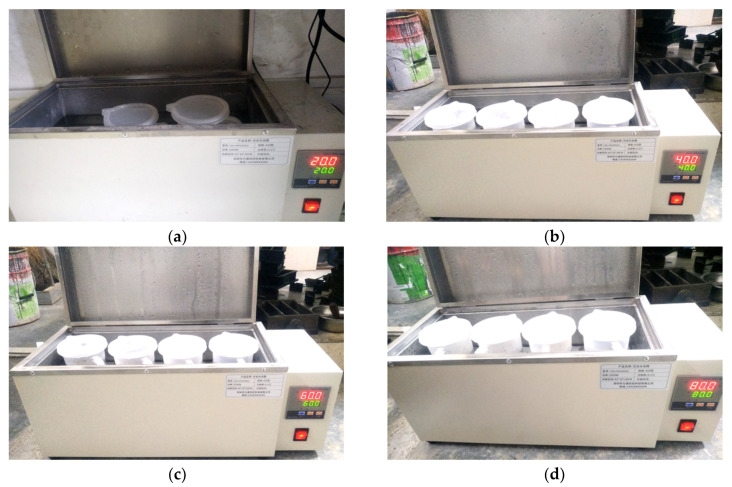
Experimental materials preheating. (**a**) Preheating temperature is 20 °C; (**b**) preheating temperature is 40 °C. (**c**) preheating temperature is 60 °C; (**d**) preheating temperature is 80 °C.

(1) All kinds of experimental materials needed were weighed, put into containers, respectively, and sealed, so as to prevent the experimental materials from getting damp in the process of preheating. At the constant temperature conditions of 20, 40, 60 and 80 °C, the experimental materials were preheated by electric constant temperature water bath (HX-HH420 water bath box, Shenzhen, China) for 24 h, as shown in Figure 5.
(2) According to the commonly used slurry ratio in grouting engineering, the water:cement ratio of the ordinary cement slurry was 0.6:1, and the mixing volume ratio of cement slurry and sodium silicate slurry in the cement-sodium silicate slurry (volume of cement slurry:volume of sodium silicate slurry) was 5:1. The mixing volume ratio of slurry A and slurry B in the HGTGS (volume of slurry A:volume of slurry B) was 5:1.
(3) The experimental materials at different temperatures were mixed in the beaker according to the above proportion and stirred evenly. The ordinary cement slurry, the cement-sodium silicate slurry and the HGTGS in constant temperature conditions of 20, 40, 60 and 80 °C were prepared, respectively. Immediately take the 350 mL tested slurry and place it in the measuring cup of 500 mL.
(4) The measuring cup was placed directly below the SNB-2 microcomputer digital display viscometer, the lifting knob of the viscometer was slowly adjusted, and the height of the rotor in the tested slurry was adjusted until the liquid level mark of the rotor (middle of the groove) was level with the slurry surface. The viscosity was tested when the turning speed of the viscometer rotor was 3 r/min. The viscosity and temperature of the tested slurry can be displayed on the display screen of the viscometer in real-time, and the tested data can be recorded automatically. For the ordinary cement slurry, the viscosity of the sample was recorded once every 10 min. For the cement-sodium silicate slurry, the viscosity of the sample was recorded once every 2.5 s. For the HGTGS, the viscosity of the sample was recorded once every 10 s.
(5) For each slurry in various temperature conditions, three sets of repeated experiments were performed, and the average value of the three sets of results was obtained as the viscosity value of the slurry.

## 3. Results

### 3.1. Experimental Curve of the Time-Varying Viscosity of Slurry in Different Temperature Conditions

**Figure 6:**
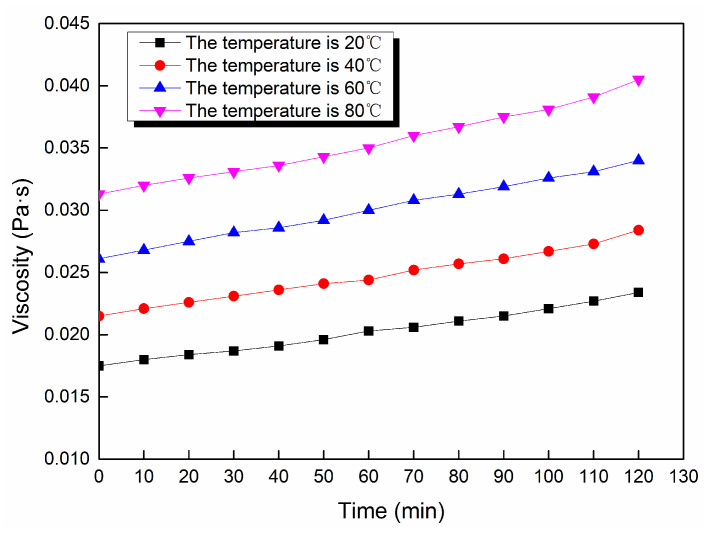
The time-varying viscosity curve of the ordinary cement slurry at different temperatures.

**Figure 7:**
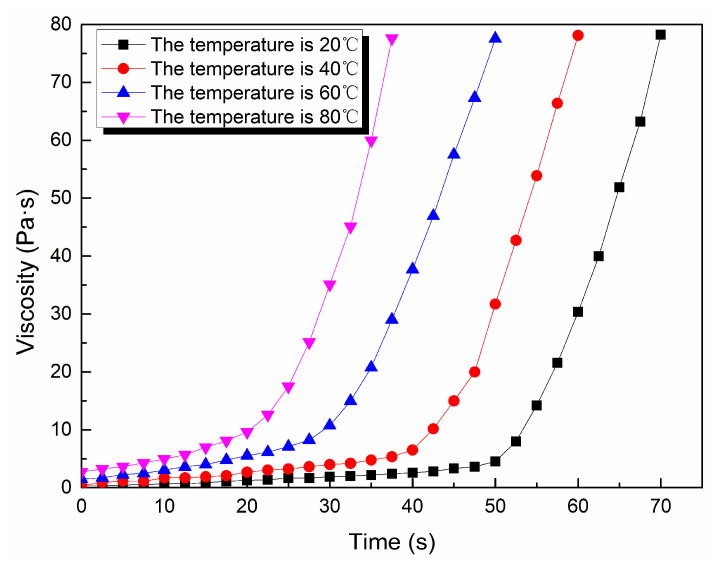
The time-varying viscosity curve of the cement-sodium silicate slurry at different temperatures.

**Figure 8:**
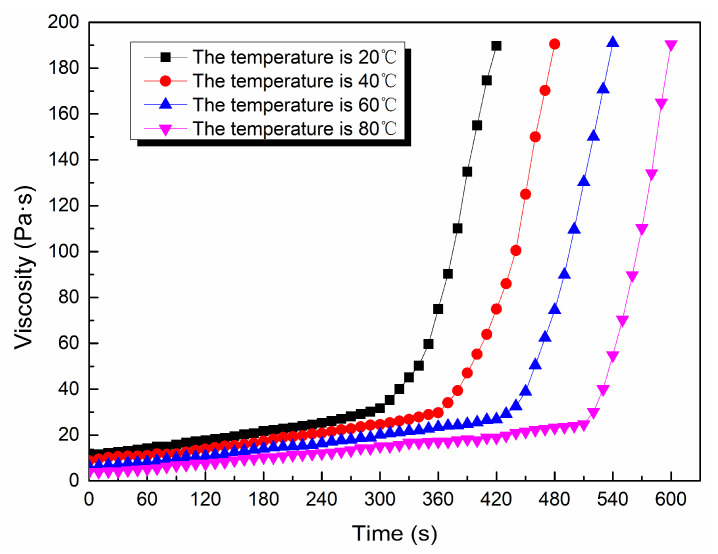
The time-varying viscosity curve of the HGTGS at different temperatures.

Through the viscosity measurement experiment, the viscosity data of the ordinary cement slurry, the cement-sodium silicate slurry and the HGTGS with specific proportions at 20, 40, 60 and 80 °C were obtained. As a result, the viscosity curves of the three kinds of slurry with time in different temperature conditions were obtained. Figure 6 shows the time-varying viscosity curve of the ordinary cement slurry with a water:cement ratio of 0.6:1 at different temperatures. Figure 7 shows the time-varying viscosity curve of the cement-sodium silicate slurry at different temperatures when the water:cement ratio of cement slurry was 0.6:1 and the volume ratio of cement slurry and sodium silicate slurry was 5:1. Figure 8 shows the time-varying viscosity curve of the HGTGS at different temperatures when the volume ratio of slurry A and slurry B was 5:1.

### 3.2. Analysis of the Time-Varying Laws of Slurry Viscosity in Specific Temperature Conditions

**Figure 9:**
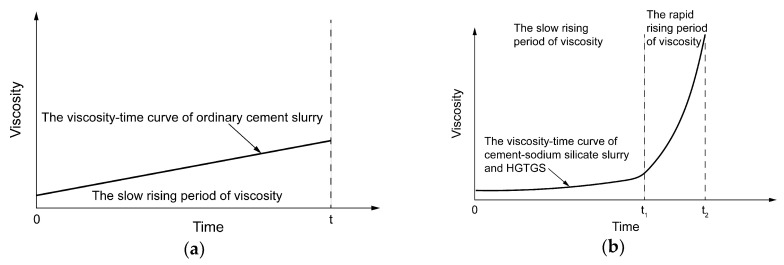
Schematic diagram of the division of viscosity periods of the ordinary cement slurry, the cement-sodium silicate slurry and the HGTGS at a specific temperature. (**a**) Schematic diagram of the division of viscosity periods of ordinary cement slurry; (**b**) schematic diagram of the division of viscosity periods of the cement-sodium silicate slurry and the HGTGS.

As shown in Figure 9a, the viscosity of the ordinary cement slurry has a time-varying characteristic at a specific temperature, but the viscosity of the slurry increases slowly because of the long hydration reaction time of the cement slurry. According to the change law of slurry viscosity with time in specific temperature conditions, the process of viscosity change of the ordinary cement slurry only includes one stage of the slow rising period of viscosity. With the increase of hydration time, the viscosity of ordinary cement slurry will gradually increase until it is solidified.

As shown in Figure 9b, the viscosity changes of the cement-sodium silicate slurry and the HGTGS had obvious time-varying characteristics at a specific temperature. The viscosity of the slurry increased with the increase of time, and the viscosity of the slurry had a sudden increase trend after a certain period of time. According to the change law of slurry viscosity with time in specific temperature conditions, taking the moment when the viscosity suddenly increases as the boundary, the process of viscosity changes of cement-sodium silicate slurry and the HGTGS can be divided into two stages: The slow rising period of viscosity and the rapid rising period of viscosity. The period before the moment when the viscosity suddenly increases is the induction period of the gel reaction of the slurry, the gel reaction rate of the slurry is slow, its viscosity increases slowly and its viscosity value is low. The period after the moment when the viscosity suddenly increases is the acceleration period of the gel reaction of the slurry, the gel reaction rate of the slurry is accelerated, the viscosity of the slurry increases rapidly, and the slurry thickens rapidly. At this time, the slurry presents a paste of solid-liquid mixture, but it still has the properties of flow and diffusion, until the slurry is condensed and solidified, whereby the slurry will lose its fluidity.

According to the viscosity time-varying characteristics of three different slurries in specific temperature conditions obtained by viscosity experiments, the parameters of viscosity periods of slurries were obtained after dividing the reaction process of different types of slurries into two stages: The slow rising period of viscosity and the rapid rising period of viscosity, which can more intuitively and conveniently show the characteristics of three different slurries in the slow rising period of viscosity and the rapid rising period of viscosity in specific temperature conditions. In the actual grouting construction, according to the on-site geological environment and the parameters of viscosity periods of different types of slurries, the suitable slurry can be selected to achieve the best grouting treatment effect. The parameters of viscosity periods of the ordinary cement slurry, the cement-sodium silicate slurry and the HGTGS in specific temperature conditions are shown in Table 5.

By comparing and analyzing the above data and the viscosity curves of the slurry, the following conclusions could be drawn:

(1) The viscosity of the ordinary cement slurry increases with the increase of temperature. The ordinary cement slurry can delay the gel and prolong the slow rising period of viscosity at a lower temperature.
(2) The viscosity of the cement-sodium silicate slurry increases with the increase of temperature. The cement-sodium silicate slurry can delay the gel and prolong the slow rising period of viscosity at a lower temperature.
(3) The viscosity of the HGTGS decreases with the increase of temperature. The HGTGS can delay the gel and prolong the slow rising period of viscosity at a higher temperature.

### 3.3. Fitting Analysis of Experimental Data of Slurry Viscosity in Different Temperature Conditions

**Figure 10:**
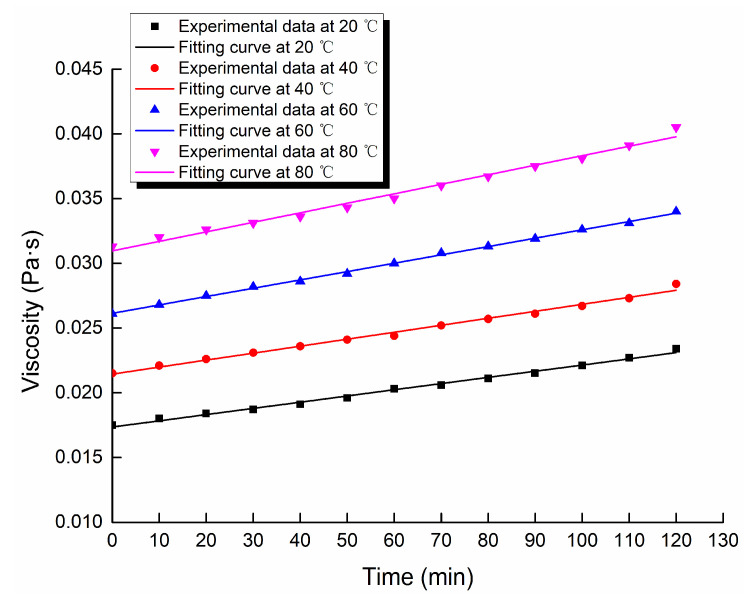
Fitting curves of viscosity of the ordinary cement slurry.

**Figure 11:**
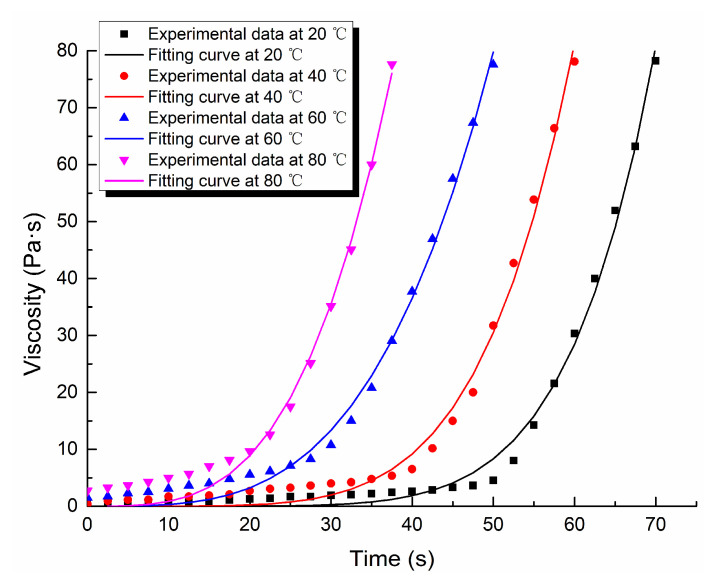
Fitting curves of viscosity of the cement-sodium silicate slurry.

**Figure 12:**
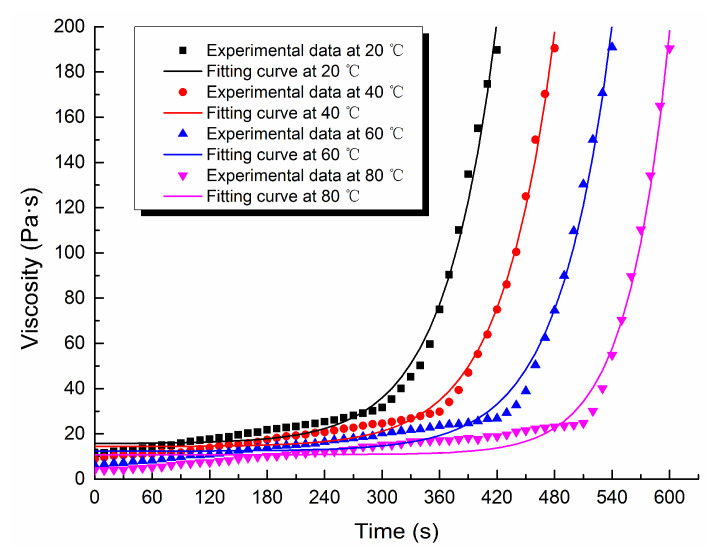
Fitting curves of viscosity of the HGTGS.

The existing viscosity models mainly reflect the time-varying characteristics of slurry viscosity at room temperature, but rarely reflect the time-varying characteristics of slurry viscosity at high temperature. In the study, considering the effect of temperature on the development of slurry viscosity, the function fitting method was used to fit and analyze the time-varying viscosity data of the ordinary cement slurry, the cement-sodium silicate slurry and the HGTGS in different temperature conditions. The viscosity equations of three different slurries at different temperatures obtained by fitting are shown in Table 6. The viscosity function curves of slurries obtained by fitting are shown in Figure 10, Figure 11 and Figure 12. They can better reflect the viscosity time-varying characteristics of different slurries at different temperatures, and are the supplement and development of the existing viscosity models.

The following conclusions can be drawn from the experimental data fitting of the three slurries in different temperature conditions:

(1) The viscosity equations of the ordinary cement slurry, the cement-sodium silicate slurry and the HGTGS in different temperature conditions obtained by function fitting can better reflect the time-varying characteristics of the viscosity of the slurries, which can be used for theoretical calculation and numerical simulation of grouting in high ground temperature environments.
(2) There are large differences in the time-varying characteristics of the viscosity of the ordinary cement slurry, the cement-sodium silicate slurry and the HGTGS in specific temperature conditions. The time-varying function of the viscosity of the ordinary cement slurry is suitable for fitting with a linear function, the time-varying function of the viscosity of the cement-sodium silicate slurry is suitable for fitting with a power law function, and the time-varying function of the viscosity of the HGTGS is suitable for fitting with an exponential function.
(3) The time-varying equations of the viscosity of a specific slurry in different temperature conditions are consistent in form, indicating that temperature changes do not affect the time-varying law of the viscosity of the slurry, but only affect the viscosity value of the slurry.
(4) The existing typical rheological models include the Newton model, the Bingham model and the Herschel-Bulkley model. The Newton model is applied to describe the rheological properties of a few thinning cement slurries. The Bingham model is applied to describe the rheological properties of most cement-based composite slurries. The Herschel-Bulkley model is applied to describe the rheological properties of a few chemical slurries with high viscosity. When the flow pattern of the slurry is determined by the rheological experiment, and the viscosity equation obtained in the study is substituted into the slurry rheological model, the slurry rheological equation and grouting diffusion model considering the time-varying characteristic of viscosity can be obtained.

### 3.4. Flow Pattern and Variation of Rheological Parameters of Slurry in Different Temperature Conditions

**Figure 13:**
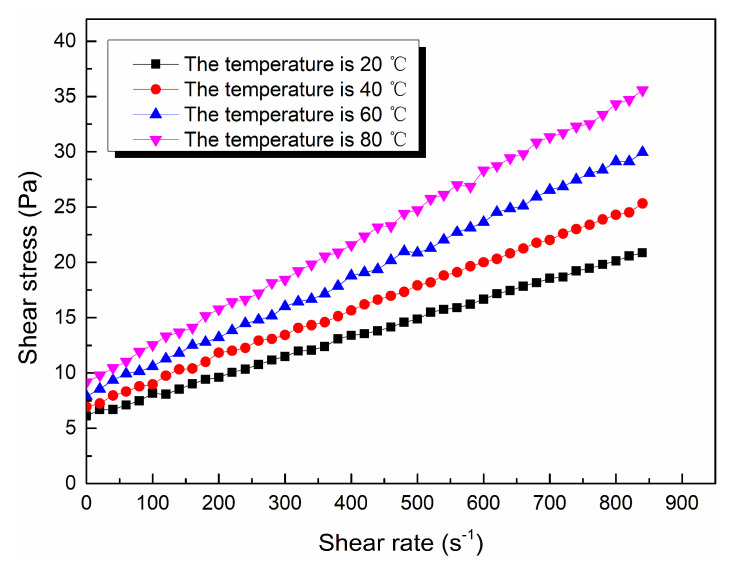
The rheological curve of the ordinary cement slurry at different temperatures.

**Figure 14:**
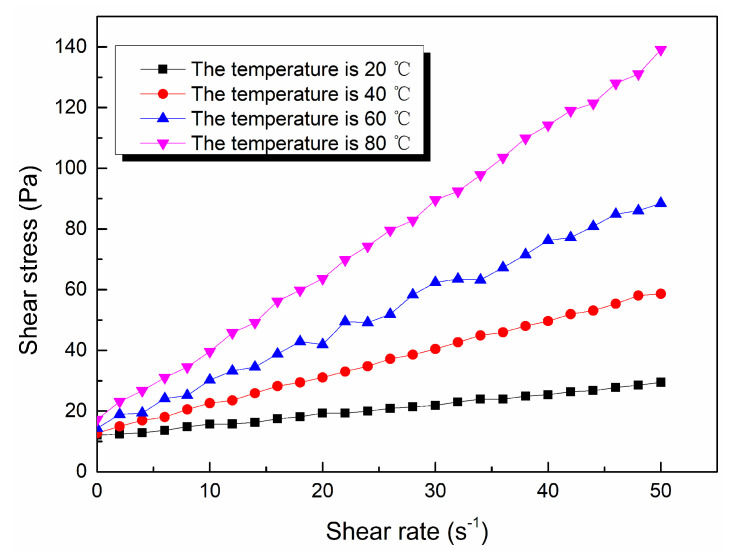
The rheological curve of the cement-sodium silicate slurry at different temperatures.

**Figure 15:**
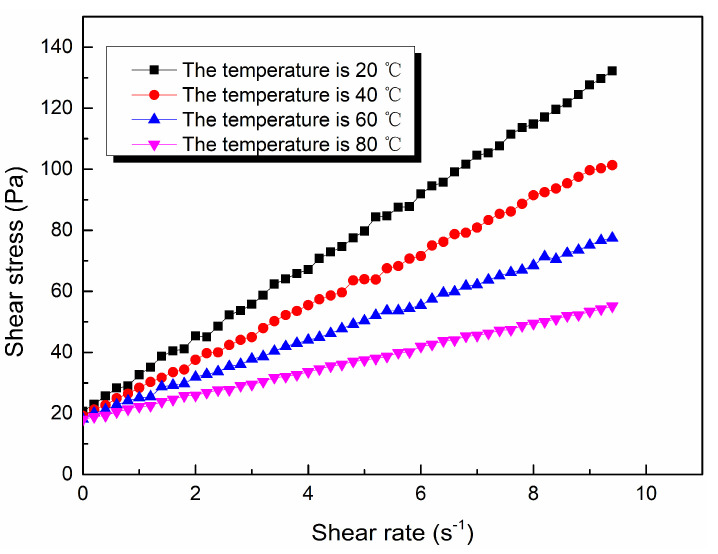
The rheological curve of the HGTGS at different temperatures.

The flow pattern of slurry is the basis for the study of a grouting diffusion model. In this experiment, the rheological curves of the ordinary cement slurry, the cement-sodium silicate slurry and the HGTGS with specific proportions at 20, 40, 60 and 80 °C were obtained. In addition, the rheological equations of all kinds of slurries were obtained by the function fitting method, based on which the flow patterns of three kinds of slurries in different temperature conditions were obtained. The rheological curves of three different slurries at different temperatures are shown in Figure 13, Figure 14 and Figure 15. The rheological equations of three different slurries and their flow patterns at different temperatures are shown in Table 7.

**Figure 16:**
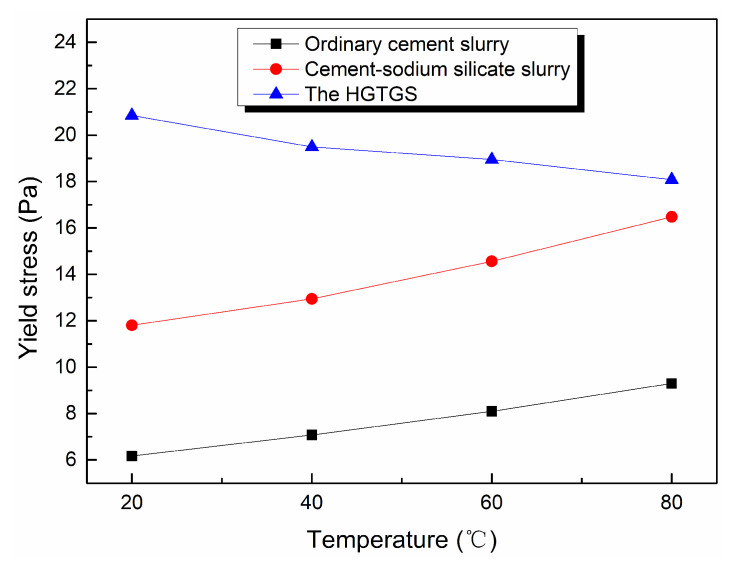
The variation of yield stress of different types of slurries with temperature.

The temperature had an effect on the rheological parameters of slurry. The variation of yield stress of the ordinary cement slurry, the cement-sodium silicate slurry and the HGTGS with temperature is shown in Figure 16. 

By comparing and analyzing the above experimental results, the following conclusions could be drawn:

(1) In different temperature conditions, the flow pattern of the ordinary cement slurry remains unchanged, and its flow pattern is Bingham fluid. At the same shear rate, the shear stress of the ordinary cement slurry increases with the increase of temperature. The yield stress of the ordinary cement slurry can be regarded as a linear increase with the increase of temperature.
(2) In different temperature conditions, the flow pattern of the cement-sodium silicate slurry remains unchanged, and its flow pattern is Bingham fluid. At the same shear rate, the shear stress of the cement-sodium silicate slurry increases with the increase of temperature. The yield stress of the cement-sodium silicate slurry can be regarded as a linear increase with the increase of temperature.
(3) In different temperature conditions, the flow pattern of the HGTGS remains unchanged, and its flow pattern is Bingham fluid. At the same shear rate, the shear stress of the HGTGS decreases with the increase of temperature. The yield stress of the HGTGS can be regarded as a linear decrease with the increase of temperature.

## 4. Discussion

### 4.1. Analysis of Viscosity Characteristics of Grouting Slurry at High Ground Temperature

**Figure 17:**
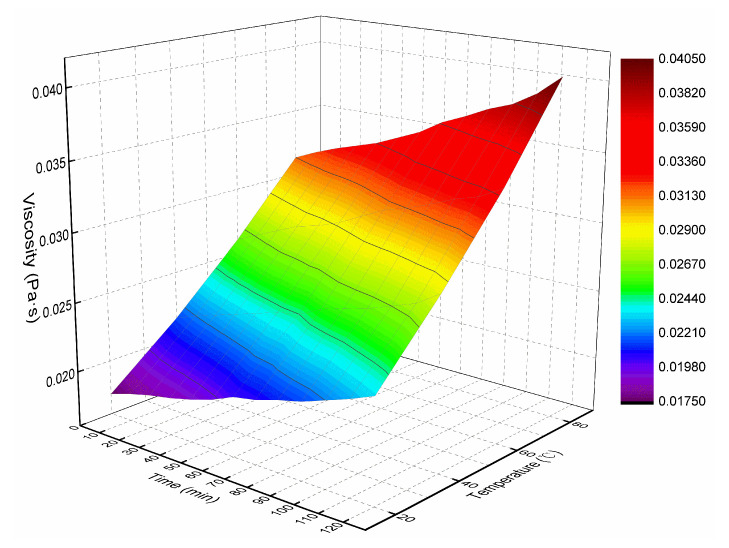
The relationship of viscosity-temperature-time of the ordinary cement slurry.

**Figure 18:**
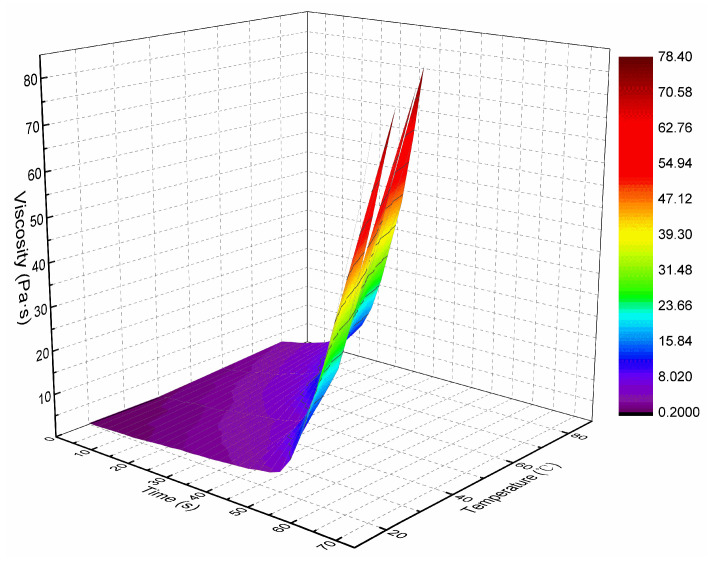
The relationship of viscosity-temperature-time of the cement-sodium silicate slurry.

**Figure 19:**
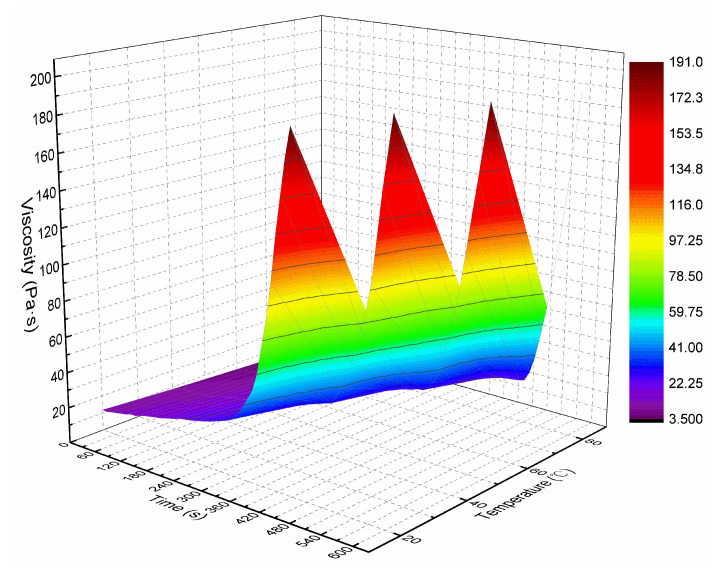
The relationship of viscosity-temperature-time of the HGTGS.

The viscosity of the slurry represents the rheological property of the slurry, which is the internal friction caused by the flow of the slurry. It not only directly affects the diffusion distance of slurry, but also affects grouting parameters such as grouting pressure, grouting flow and so on. Temperature and time had a very obvious influence on the viscosity of grouting slurry. The increase of temperature had an increasing effect on the viscosity of the ordinary cement slurry and the cement-sodium silicate slurry, and had a weakening effect on the viscosity of the HGTGS. The increase of time had an increasing effect on the viscosity of the ordinary cement slurry, the cement-sodium silicate slurry and the HGTGS. The ambient temperature of high ground temperature geology is often between 30–80 °C. When grouting in high ground temperature environment, the transport process of slurry is the process of slurry being heated step-by-step, so the temperature-time variation characteristics of slurry viscosity must be considered. The relationship surface of viscosity-temperature-time of the ordinary cement slurry is shown in Figure 17. The relationship surface of viscosity-temperature-time of the cement-sodium silicate slurry is shown in Figure 18. The relationship surface of viscosity-temperature-time of the HGTGS is shown in Figure 19. 

It can be seen from Figure 13, Figure 14 and Figure 15 that, considering both temperature and time effects, the viscosity equation of grouting slurry can be expressed as:

(1) μ=μ(t,T)

where *t* is time; *T* is temperature.

In this study, the function fitting method was used to obtain the quantitative relationship among viscosity, time and temperature of different types of slurries, as shown in Table 8.

It can be seen from Table 4 that there are significant differences in the viscosity characteristics of different types of slurries at high ground temperature, and the equations obtained by fitting can better reflect the viscosity characteristics of the slurries at high ground temperature.

The viscosity evolution of grouting slurry at high ground temperature is an extremely complicated process. Equation (1) can be used as a unified description equation for the viscosity characteristics of grouting slurry at high ground temperature.

### 4.2. Analysis of Rheological Characteristics of Grouting Slurry at High Ground Temperature

Most cement-based grouting materials are typical fluids with yield stress. When the slurry is subjected to shear, there are two opposite effects in the slurry, which will lead to shear thinning and shear thickening of the slurry, respectively. Shear thinning refers to the phenomenon that the viscosity of slurry decreases gradually with the increase of shear rate, on the contrary, it is shear thickening. The main reason for the shear thinning of grouting materials is that the reticular flocculation structure in the slurry is gradually destroye by the shearing action, resulting in the decrease of the viscosity of the slurry. The main reason for the shear thickening of grouting materials is that at a certain shear rate, the solid particles in the slurry overcome the repulsive force between the particles and temporarily gather into particle clusters, which lead to the shear thickening of the slurry. In addition, when the shear rate is low, the particles in the slurry are in an orderly distribution state, which leads to the low viscosity of the slurry. When the shear rate is high, the particles in the slurry change into disordered distribution, which leads to the increase of the viscosity of slurry and the shear thickening of the slurry. Therefore, for on-site grouting, the freshly mixed slurry should be injected into the grouted medium as soon as possible, otherwise, shear thickening of the slurry will appear due to power stirring (equivalent to a relatively high shear rate), resulting in the increase of the viscosity of slurry, which will increase the resistance of the slurry into the grouted medium and affect the diffusion range of the slurry.

Because the factors that lead to shear thinning and shear thickening of the slurry exist at the same time, the rheological characteristics of grouting materials are the result of the interaction between them. When the effect of factors leading to the shear thinning of slurry is strong, shear thinning of the slurry appears, on the contrary, shear thickening of the slurry appears. When the two effects are roughly the same and cancel each other out, the grouting material can be regarded as Bingham fluid. 

The rheological characteristics of grouting material can be characterized by its rheological model. In this study, the shear stress of the ordinary cement slurry, the cement-sodium silicate slurry and the HGTGS increases linearly with the increase of shear rate at different temperatures, and the flow patterns of the three types of slurries are Bingham model. The mathematical expression of the Bingham model is as follows:

(2) $$\tau =\tau_{0}+\mu \gamma$$

where τ is shear stress (Pa); *τ_0_* is yield stress (Pa); *μ* is viscosity (Pa·s); *γ* is shear rate (s^−1^).

It can be seen that, from the rheological equation of Bingham fluid, the viscosity is the slope of the rheological curve of Bingham fluid. The rheological curves of different types of slurries at different temperatures are basically linear, so the viscosity of the three slurries is fixed, that is, the viscosity of the slurries does not change with the change of shear rate. This is because the effect of the factors that lead to the shear thinning of slurry is equal to that of the factors leading to the shear thickening of slurry, and counteract each other, which shows that the viscosity of slurry is not affected by shear rate macroscopically. Therefore, for the Bingham slurry in this study, the effect of shear rate on the viscosity of slurry cannot be considered.

Temperature and time have a very obvious effect on the viscosity of slurry. The change of yield stress of slurry with temperature can be regarded as linear. Existing studies have shown that yield stress of slurry has no time-varying characteristics [23,24].

According to the above analysis, the rheological model of grouting slurry in specific temperature conditions can be expressed as follows:

(3) $$\tau =\tau_{0}+\mu (t)\gamma$$

where *μ(t)* is the viscosity equation of slurry considering time-varying characteristics. The viscosity equation of the ordinary cement slurry is in the form of linear function, the viscosity equation of the cement-sodium silicate slurry is in the form of power-law function, and the viscosity equation of the HGTGS is in the form of exponential function.

When the temperature effect and time effect are considered at the same time, the rheological model of grouting slurry can be expressed as follows:

(4) $$\tau =\tau_{0}(T)+\mu (t,T)\gamma$$

where *τ_0_*(*T*) is a temperature-dependent coefficient, and it has no time-varying characteristics; *μ*(*t,T*) is the viscosity equation of slurry considering temperature-varying and time-varying characteristics.

The rheological model of slurry considering both temperature effect and time effect (Equation (4)) can be used to characterize the rheological characteristics of grouting slurry in a high ground temperature environment.

### 4.3. Applicability Analysis of Different Types of Grouting Materials

The viscosity characteristics of the ordinary cement slurry, the cement-sodium silicate slurry and the HGTGS are significantly different. In the actual grouting engineering, the slurry selection should be carried out according to the field geological environment and the characteristics of grouting materials, and reasonable grouting technology should be adopted to achieve the best grouting treatment effect.

Generally speaking, in order to achieve a good grouting effect during field grouting, the slurry is required to have two characteristics: (1) In the process of grouting, the slurry can maintain the fluidity of the slurry in the grouted medium, that is, the viscosity of the slurry is low and the viscosity increases little. This characteristic corresponds to the slow rising period of slurry viscosity; (2) after the end of grouting, the viscosity of the slurry increases rapidly, the slurry loses its fluidity and a stone body is formed quickly, that is, the viscosity of the slurry has the characteristic of “right-angle thickening”. This characteristic corresponds to the rapid rising period of slurry viscosity. Characteristic (1) is conducive to the full filling of the slurry into the grouted rock and soil; and characteristic (2) is conducive to the bonding of the slurry to the rock and soil, preventing the slurry from being eroded by groundwater before solidification, which can ensure that the slurry stops diffusing when it reaches a limited distance in the process of grouting, so that it will not cause excessive loss of the slurry.

The process of viscosity change of the ordinary cement slurry only includes one stage of the slow rising period of viscosity. The increasing rate of slurry viscosity is slow and the viscosity value is low, which makes the slurry have a strong ability of migration and diffusion, but weak anti-dispersion ability, so the scouring and dilution of slurry facing dynamic water should be avoided in the process of grouting. Therefore, the ordinary cement slurry is suitable for grouting reinforcement engineering in anhydrous or static water conditions.

The process of viscosity change of the cement-sodium silicate slurry includes two stages: The slow rising period of viscosity and the rapid rising period of viscosity. During the slow rising period of viscosity, the viscosity of the slurry increases slowly and the viscosity value is low, which can be used for migration and diffusion. During the rapid rising period of viscosity, the viscosity of the slurry rises rapidly, which is the key stage of plugging the gushing water. In addition, the cement-sodium silicate slurry can delay the gel and prolong the slow rising period of viscosity at a lower temperature, which is beneficial to ensure the diffusion range of the slurry in the grouted medium. Therefore, the cement-sodium silicate slurry is suitable for the plugging engineering of dynamic water grouting in a normal temperature environment.

The process of viscosity change of the HGTGS also includes two stages: The slow rising period of viscosity and the rapid rising period of viscosity. During the slow rising period of viscosity, the viscosity of the slurry increases slowly and has a certain degree of anti-dispersion, maintaining flow and diffusion. During the rapid rising period of viscosity, the slurry quickly gelled, blocking water inrush. In addition, the HGTGS can delay the gel and prolong the slow rising period of viscosity at a higher temperature, which ensures the diffusion range of the slurry in the grouted medium. Therefore, the HGTGS is suitable for the plugging engineering of dynamic water grouting in a high ground temperature environment. 

## 5. Conclusions

In order to study the viscosity characteristics of grouting slurry in a high ground temperature environment, in this paper, using the ordinary cement slurry, the cement-sodium silicate slurry and the HGTGS, the viscosity experiments of different types of slurry at different preheating temperatures were carried out and the experimental data were fitted and analyzed. The following main conclusions can be drawn:

(1) In certain temperature conditions, there are significant differences in the time-varying characteristics of the viscosity of the ordinary cement slurry, the cement-sodium silicate slurry and the HGTGS. The process of viscosity change of the ordinary cement slurry only includes one stage of the slow rising period of viscosity, while the process of viscosity change of the cement-sodium silicate slurry and the HGTGS includes two stages: The slow rising period of viscosity and the rapid rising period of viscosity. The slow rising period of viscosity can be used for the migration and diffusion of slurry, and the rapid rising period of viscosity is the key stage for grouting to block water gushing.
(2) Temperature has a significant effect on the viscosity of the slurry. The viscosity of the ordinary cement slurry and the cement-sodium silicate slurry increases with the increase of temperature, while the viscosity of the HGTGS decreases with the increase of temperature. The ordinary cement slurry and the cement-sodium silicate slurry can delay the gel and prolong the slow rising period of viscosity at lower temperature, while the HGTGS can delay the gel and prolong the slow rising period of viscosity at higher temperature.
(3) The time-varying equation of viscosity of a specific slurry in different temperature conditions is consistent in form. The time-varying equation of the viscosity of the ordinary cement slurry conforms to the linear function form, the time-varying equation of the viscosity of the cement-sodium silicate slurry conforms to the power-law function form, and the time-varying equation of the viscosity of the HGTGS conforms to the exponential function form. The time-varying equations of the viscosity of the three kinds of slurry can better reflect the characteristics of the viscosity change of the slurry, which can be used for theoretical calculation and numerical simulation of grouting in high ground temperature environment.
(4) In different temperature conditions, the flow patterns of the ordinary cement slurry, the cement-sodium silicate slurry and the HGTGS remain unchanged, and their flow patterns are Bingham fluid. At the same shear rate, the shear stress of the ordinary cement slurry and the cement-sodium silicate slurry increases with the increase of temperature, while the shear stress of the HGTGS decreases with the increase of temperature. The yield stress of the ordinary cement slurry and the cement-sodium silicate slurry can be regarded as a linear increase with the increase of temperature, while the yield stress of the HGTGS can be regarded as a linear decrease with the increase of temperature.
(5) The viscosity evolution of grouting slurry in a high ground temperature environment is an extremely complicated process. The viscosity equation of slurry that considers both temperature and time effects can be used as a unified description equation for the viscosity characteristics of grouting slurry in a high ground temperature environment. The rheological model of slurry considering both temperature effect and time effect can be used to characterize the rheological characteristics of grouting slurry in a high ground temperature environment.
(6) There are great differences in the engineering applicability of the ordinary cement slurry, the cement-sodium silicate slurry and the HGTGS. The ordinary cement slurry is suitable for grouting reinforcement engineering in anhydrous or static water conditions, the cement-sodium silicate slurry is suitable for the plugging engineering of dynamic water grouting in a normal temperature environment, and the HGTGS is suitable for the plugging engineering of dynamic water grouting in a high ground temperature environment.

## Figures and Tables

**Table 1:** The major chemical constituents and physical properties of the cement.

Chemical Constituents	CaO	SiO_2_	Al_2_O_3_	MgO	SiO_3_	Fe_2_O_3_	Loss on Ignition
Content (%)	62.89	19.96	4.35	4.70	3.30	3.50	1.30
Physical Properties	Initial Setting Time	Final Setting Time	3 days Rupture Strength	28 days Rupture Strength	3 days Compressive Strength	28 days Compressive Strength	Density
Test Results	135 min	186 min	6.3 MPa	8.2 MPa	38.7 MPa	56.0 MPa	3.14 g/cm^3^

**Table 2:** The chemical compositions of the HGTGS.

Chemical Compositions	Group Agent A				Group Agent B	
	Cement	Bentonite	Slag	Water	Sodium Silicate	Modified Additives
Content	The Amount of Cement Needed for the Experiment	10% of the Quality of Cement	20% of the Quality of Cement	Water:Solid = 0.6:1	The Amount of Sodium Silicate Needed for the Experiment	0.3% of the Quality of Sodium Silicate

Group agent A and group agent B were adopted at 5:1 according to the mixed volume ratio (group agent A:group agent B).

**Table 3:** The density, setting time, stone rate and bleeding rate of the HGTGS in different temperature conditions.

Temperature (°C)	20 °C	40 °C	60 °C	80 °C
Density (g/cm^3^)	1.496	1.678	1.741	1.786
Initial Setting Time (s)	330	380	450	540
Final Setting Time (s)	490	520	570	630
Stone Rate (%)	96.4	97.3	98.4	99.8
Bleeding Rate (%)	3.6	2.8	1.9	0.8

**Table 4:** The compressive strength and rupture strength of stone body of the HGTGS at different ages in different temperature conditions.

Age (days)	20 °C		40 °C		60 °C		80 °C	
	Compressive Strength (MPa)	Rupture Strength (MPa)	Compressive Strength (MPa)	Rupture Strength (MPa)	Compressive Strength (MPa)	Rupture Strength (MPa)	Compressive Strength (MPa)	Rupture Strength (MPa)
3	4.30	1.02	6.90	1.44	7.60	1.68	8.10	1.80
7	5.32	1.45	8.02	1.93	8.58	1.98	7.85	1.74
28	7.42	1.89	9.3	2.21	9.71	2.57	7.25	1.63
56	8.98	2.29	10.88	2.59	10.96	2.78	6.90	1.57
90	9.61	2.45	11.26	2.68	11.54	2.97	7.03	1.58

**Table 5:** The parameters of viscosity periods of three different slurries in specific temperature conditions.

Type of Slurry	Water:Cement Ratio of Cement Slurry	Mixing Volume Ratio of Slurry	Temperature (°C)	The Slow Rising Period of Viscosity (s)	The Rapid Rising Period of Viscosity (s)
Ordinary Cement Slurry	0.6:1	-	20	>7200	-
			40	>7200	-
			60	>7200	-
			80	>7200	-
Cement-Sodium Silicate Slurry	0.6:1	5:1	20	0−50	50−70
			40	0−40	40−60
			60	0−30	30−50
			80	0−20	20−38
The HGTGS	-	5:1	20	0−300	300−420
			40	0−360	360−480
			60	0−420	420−540
			80	0−510	510−600

**Table 6:** The time-varying characteristic equations of viscosity of three kinds of slurries in different temperature conditions.

Type of Slurry	Temperature (°C)	The Time-Varying Characteristic Equations of Viscosity	R^2^
Ordinary Cement Slurry	20	$\mu =0.01736+4.78571\times 10^{-5}t$	0.993
	40	$\mu =0.02144+5.39011\times 10^{-5}t$	0.991
	60	$\mu =0.02614+6.45055\times 10^{-5}t$	0.998
	80	$\mu =0.03096+7.35165\times 10^{-5}t$	0.987
Cement-Sodium Silicate Slurry	20	$\mu =2.71354\times 10^{-11}t^{6.76022}$	0.993
	40	$\mu =2.18059\times 10^{-8}t^{5.38292}$	0.990
	60	$\mu =8.93584\times 10^{-5}t^{3.50253}$	0.991
	80	$\mu =3.21232\times 10^{-4}t^{3.4143}$	0.986
The HGTGS	20	$\mu =15.69564+0.07822e^{0.01852t}$	0.989
	40	$\mu =14.32758+0.02873e^{0.01825t}$	0.992
	60	$\mu =12.4065+0.00921e^{0.01838t}$	0.987
	80	$\mu =10.83789+0.000179456e^{0.0231t}$	0.983

*μ* is viscosity, *t* is time, R^2^ is the square of the correlation coefficient.

**Table 7:** The rheological equations of three kinds of slurries and their flow patterns at different temperatures.

Type of Slurry	Temperature (°C)	Yield Stress (Pa)	Rheological Equation	Flow Pattern of Slurry
Ordinary Cement Slurry	20	6.170	τ=6.170+0.0176γ	Bingham Fluid
	40	7.079	τ=7.079+0.0215γ	
	60	8.098	τ=8.098+0.0260γ	
	80	9.303	τ=9.303+0.0311γ	
Cement-Sodium Silicate Slurry	20	11.807	τ=11.807+0.3461γ	Bingham Fluid
	40	12.940	τ=12.940+0.9232γ	
	60	14.563	τ=14.563+1.5006γ	
	80	16.477	τ=16.477+2.4166γ	
The HGTGS	20	20.851	τ=20.851+11.8111γ	Bingham Fluid
	40	19.492	τ=19.492+8.8472γ	
	60	18.947	τ=18.947+6.2322γ	
	80	18.080	τ=18.080+3.9167γ	

*τ* is shear stress, *γ* is shear rate.

**Table 8:** The viscosity equations of different types of slurries at high ground temperature.

Type of Slurry	Viscosity Equations	R^2^
Ordinary Cement Slurry	$\mu =0.01129+5.99451\times 10^{-5}t+2.53731\times 10^{-4}T$	0.993
Cement-Sodium Silicate Slurry	$\mu =32.60012-2.63537t-0.89108T+0.03752t^{2}+0.00595T^{2}+0.03487tT$	0.899
The HGTGS	$\mu =\frac{8.49063+0.16044t-0.52721T+0.02387T^{2}-2.22531\times 10^{-4}T^{3}}{1+7.09224\times 10^{-4}t-1.3154\times 10^{-6}t^{2}-1.74557\times 10^{-8}t^{3}+0.02449T+2.10479\times 10^{-4}T^{2}}$	0.960

## References

[1] Hu Y.P., Wang M.N., Wang Q.L., Liu D.G., Tong J.J. (2019). Field test of thermal environment and thermal adaptation of workers in high geothermal tunnel. Build. Environ..

[2] Liu P., Cui S.G., Li Z.H., Xu X.F., Guo C. (2019). Influence of surrounding rock temperature on mechanical property and pore structure of concrete for shotcrete use in a hot-dry environment of high-temperature geothermal tunnel. Constr. Build. Mater..

[3] Li Y.Q., Lu L.C., Wang S.D., Wang H. (2010). Influence of curing temperature on performance of belite-barium calcium sulphoaluminate cement. Bull. Chin. Ceram. Soc..

[4] Termkhajornkit P., Barbarulo R. (2012). Modeling the coupled effects of temperature and fineness of Portland cement on the hydration kinetics in cement paste. Cem. Concr. Res..

[5] Zakoutsky J., Tydlitat V., Cerny R. (2012). Effect of temperature on the early-stage hydration characteristics of Portland cement: A large-volume calorimetric study. Constr. Build. Mater..

[6] Kumar M., Singh S.K., Singh N.P. (2012). Heat evolution during the hydration of Portland cement in the presence of fly ash, calcium hydroxide and super plasticizer. Thermochim. Acta.

[7] Gallucci E., Zhang X., Scrivener K.L. (2013). Effect of temperature on the microstructure of calcium silicate hydrate (CSH). Cem. Concr. Res..

[8] Narmluk M., Nawa T. (2011). Effect of fly ash on the kinetics of portland cement hydration at different curing temperatures. Cem. Concr. Res..

[9] Zhang W.H., Zhang Y.S., Liu L.B., Zhang G.R., Liu Z.Y. (2012). Investigation of the influence of curing temperature and silica fume content on setting and hardening process of the blended cement paste by an improved ultrasonic apparatus. Constr. Build. Mater..

[10] Zhang Y.S., Zhang W.H., She W., Ma L.G., Zhu W.W. (2012). Ultrasound monitoring of setting and hardening process of ultra-high performance cementitious materials. NDT E Int..

[11] Shi Z.J., Fan L.D., Song Y., Yu Y.Q., Li K.F. (2018). Study on properties of cement-based grouting material in high temperature. Chin. J. Ingr. Space Eng..

[12] Wang M.N., Hu Y.P., Wang Q.L., Tian H.T., Liu D.G. (2019). A study on strength characteristics of concrete in variable temperature curing conditions in ultra-high geothermal tunnels. Constr. Build. Mater..

[13] Sajedi F., Razak H.A. (2011). Effects of curing regimes and cement fineness on the compressive strength of ordinary Portland cement mortars. Constr. Build. Mater..

[14] Al-Gahtani A.S. (2010). Effect of curing methods on the properties of plain and blended cement concretes. Constr. Build. Mater..

[15] Dai C.Q., Wang Y., Wu A.X., Qi Y., Chen Z.Q. (2019). Effect of temperature on the structure of paste slurry at low constant shear rate. Energy Sources Part A Recovery Util. Environ. Eff..

[16] Liu R.T., Li S.C., Zhang Q.S., Yuan X.S., Han W.W. (2011). Experiment and application research on a new type of dynamic water grouting material. Chin. J. Rock. Mech. Eng..

[17] Chen P., Zhang S.M., Yang H.M., Hu C. (2019). Effects of Curing Temperature on Rheological Behaviour and Compressive Strength of Cement Containing GGBFS. Wuhan Univ. Technol. Mat. Sci. Ed..

[18] Abd-El Aziz M.A., El Aleem S.A., Heikal M. (2012). Physico-chemical and mechanical characteristics of pozzolanic cement pastes and mortars hydrated at different curing temperatures. Constr. Build. Mater..

[19] Sajedi F. (2012). Effect of curing regime and temperature on the compressive strength of cement-slag mortars. Constr. Build. Mater..

[20] Zhu M.T., Zhang Q.S., Zhang X., Hui B. (2018). Comparative Study of Soil Grouting with Cement Slurry and Cement-Sodium Silicate Slurry. Adv. Mater. Sci. Eng..

[21] Yang X.D., Liu J.C. (1987). Research on Filling Ability of Cement Slurry.

[22] Du J.H., Qin M.W., Xiao R.J. (1987). Foreign Chemical Grouting Tutorial.

[23] Ruan W.J. (2005). Spreading model of grouting in rock mass fissures based on time-dependent behavior of viscosity of cement-based grouts. Chin. J. Rock. Mech. Eng..

[24] Ruan W.J. (2005). Research on diffusion of grouting and basic properties of grouts. Chin. J. Geotech. Eng..

[25] Wang H. (2019). Study on Time-dependent Viscosity of Slurry and Its Diffusion Model. Proceedings of the 5th International Conference on Energy Materials and Environment Engineering (ICEMEE).

[26] Li S.C., Liu R.T., Zhang Q.S., Sun Z.Z., Zhang X., Zhu M.T. (2013). Research on C-S slurry diffusion mechanisn with time-dependent behavior of viscosity. Chin. J. Rock. Mech. Eng..

[27] Li S.C., Han W.W., Zhang Q.S., Liu R.T., Wen X.J. (2013). Research on time-dependent behaviour of viscosity of fast curing grouts in underground construction grouting. Chin. J. Rock. Mech. Eng..

[28] Liu J., Zhang Z.S., Han Y., Wu X. (2015). Backfilled grouting diffusion law and model of pressure on segments of shield tunnel considering viscosity variation of cement grout. Rock. Soil Mech..

[29] Zhang L.Z., Zhang Q.S., Liu R.T., Li S.C., Wang H.B., Li W., Zhang S.J., Zhu G.X. (2017). Penetration grouting mechanism of quick setting slurry considering spatiotemporal variation of viscosity. Rock. Soil Mech..

[30] Zhang Q.S., Zhang L.Z., Zhang X., Liu R.T., Zhu M.T., Zheng D.Z. (2015). Grouting diffusion in a horizontal crack considering temporal and spatial variation of viscosity. Chin. J. Rock. Mech. Eng..

[31] Hou F.J., Sun K.G., Wu Q.D., Xu W.P., Ren S.J. (2019). Grout diffusion model in porous media considering the variation in viscosity with time. Adv. Mech. Eng..

[32] Zuo J.P., Hong Z.J., Xiong Z.Q., Wang C., Song H.Q. (2018). Influence of different W/C on the performances and hydration progress of dual liquid high water backfilling material. Constr. Build. Mater..

[33] Xie Y., Li J., Lu Z.Y., Jiang J., Niu Y.H. (2018). Effects of bentonite slurry on air-void structure and properties of foamed concrete. Constr. Build. Mater..

[34] Zhang W.J., Han C.H., Zhang L.Z., Wei J.C., Yin H.Y., Han W.W., Xie C., Zhou W.W. (2019). Grouting Mechanism of Cement-Based Slurry in a Concentric Annulus in High Groundwater Pressure. Adv. Civ. Eng..

[35] Assefa K.M., Kaushal D.R. (2017). A new model for the viscosity of highly concentrated multi-sized particulate Bingham slurries. Part. Sci. Technol..

[36] Liu X.Y., Cheng H., Lin J., Rong C.X., Li M.J., Xu H.D. (2019). Study of the Mechanism of Fracture Grouting in Deeply Buried Rock Strata Based on Bingham Fluid Slurry. Adv. Civ. Eng..

[37] Zhang Q.S., Zhang L.Z., Liu R.T., Li S.C., Zhang Q.Q. (2017). Grouting mechanism of quick setting slurry in rock fissure with consideration of viscosity variation with space. Tunn. Ingr. Space Technol..

[38] Campbell G.A., Zak M.E., Wetzel M.D. (2018). Newtonian, power law, and infinite shear flow characteristics of concentrated slurries using percolation theory concepts. Rheol. Acta.

[39] Blissett R.S., Rowson N.A. (2013). An empirical model for the prediction of the viscosity of slurries of coal fly ash with varying concentration and shear rate at room temperature. Fuel.

[40] Xiao J., Wang S.M., Duan X.D., Ye S.P., Wen J., Zhang Z.X. (2019). Rheological models for temperature and concentration dependencies of coal water slurry. Int. J. Coal Prep. Util..

[41] China Building Materials Academy (2007). Common Portland Cement.

